# Influence of the plant interacting entomopathogenic fungus *Beauveria bassiana* on parasitoid host choice-behavior, development, and plant defense pathways

**DOI:** 10.1371/journal.pone.0238943

**Published:** 2020-09-14

**Authors:** Rasmus Emil Jensen, Carmina Cabral, Annie Enkegaard, Tove Steenberg

**Affiliations:** Department of Agroecology, Aarhus University, Flakkebjerg, Denmark; University of Manitoba, CANADA

## Abstract

Inoculating plants with entomopathogenic fungi may influence plant nutrient uptake and growth, and herbivore performance. Knowledge is limited concerning the effects of this symbiosis on higher trophic levels. We examined how fungal treatment of faba bean seeds with the entomopathogenic fungus *Beauveria bassiana* influenced the choice-behavior and development of the aphid parasitoid *Aphidius colemani*. We also sampled plant material for analysis of changes in expression of genes related to plant defense pathways. While parasitoids were compatible with plants inoculated with *B*. *bassiana* initially (66 vs. 65% parasitization on inoculated and control plants, respectively; similar development times of parasitoids: 9.2 days), the emergence of adult parasitoids originating from aphids on fungus treated plants was significantly lower (67 vs. 76%, respectively). We also found that the defense response changed, similar to induced systemic resistance, when plants were treated with *B*. *bassiana*, similarly to what has been found for other plant symbiotic microorganisms. These novel findings show that although the application of entomopathogenic fungi to plants can alter the plants’ defense against herbivores, it may also have an impact on beneficial insects, so their function and use should be evaluated on a case-by-case basis.

## Introduction

Terrestrial plants shelter fungi that live as endophytes inside plant tissue. Endophytism has become synonymous with mutualism; however, the ecological role of endophytes can be complex and imbalanced [1, 2]. Endophytic fungi can improve growth and plant resistance to herbivores, pathogens, and various abiotic stresses and therefore may have extensive effects on populations of herbivores and their natural enemies [2–5]. While most endophyte research has focused on the obligate ascomycete grass endophytes producing toxic alkaloids, species of entomopathogenic fungi (EF), another group of hypocrealan ascomycetes, have more recently been identified as plant colonizers [6, 7]. When entomopathogenic fungi infect their arthropod host, infective spores attach to and penetrate the cuticle via chemical and mechanical force where after the fungus proliferates within its host [8]. The spores from entomopathogenic fungi are used in biocontrol of several arthropod pests as an alternative to chemical pesticides, and the fundamental aspects of the endophytic lifestyle of these fungi are of great interest to researchers and may also prove to have substantial potential for biocontrol [9–11].

The entomopathogenic fungus *Beauveria bassiana* (Balsamo-Crivelli) Vuillemin (Ascomycota: Hypocreales) has been found as a colonizing endophyte in plants of nine different families including many important crop species (e.g., maize (*Zea mays* L. (Cyperales: Poaceae)), potato (*Solanum tuberosum* L. (Solanales: Solanaceae)), soybean (*Glycine max* L. (Fabales: Fabaceae)), faba bean (*Vicia faba* L. (Fabales: Fabaceae)), and tomato (*Solanum lycopersicum* L. (Solanales: Solanaceae))) [10]. The majority of studies report positive or neutral effects on plant growth [10]. Also, most studies report a negative or neutral effect on how endophytic *B*. *bassiana* affects herbivores [12, 13], but there are reports that endophytic entomopathogenic fungi can stimulate arthropod herbivores (e.g. increased fecundity and biomass) [14–17].

Generally speaking, the consequences of microbe-plant-insect interactions are complicated and rely on many factors [18], especially when considering higher trophic levels (i.e., parasitoids and predators), and potential adverse effects between EF and natural enemies are important to consider [19, 20]. Previous studies have shown compatibility between EF and beneficial arthropods [21–23], but there are also reports of EF causing higher mortality, lower fecundity and reduced longevity in predators and parasitoids, and other studies have shown competition for resources between the two groups of natural enemies [24–26]. We know that EF may influence the performance (e.g. growth parameters and nutrient acquisition) [10, 12] and nutritional status [27, 28] of its host plant, and that choice behavior and fitness of natural enemies is influenced by their host plant and its metabolites, as well as its nutritional status [27–32]. Furthermore, several studies have reported an acquired enhanced defensive capacity in plants, which is induced by their fungal and bacterial endophytes [33, 34]. However, very few studies have explored how the treatment of plants with EF influence natural enemies and how plant responses affect these interactions. Of these, two studies found that neither endophytic *Metarhizium brunneum* Petch (Ascomycota: Hypocreales), another entomopathogenic hypocrealan ascomycete, nor *B*. *bassiana* had a negative impact on the aphid parasitoid *Aphidius colemani* Viereck (Hymenoptera: Braconidae) or the predator *Chrysoperla carnea* Stephens (Neuroptera: Chrysopidae) [35, 36]. Another study mostly found a compatible relationship between endophytic *B*. *bassiana* and two leafminer parasitoids, although some level of negative effect on the survival of the parasitoids was reported [37]. This information and the fact that previous studies on parasitoids and predators and other groups of endophytes have reported an antagonistic relationship [4, 5], makes it imperative to gain further knowledge on how the treatment of plants with EF influence natural enemies.

Plants deploy a combination of constitutive and inducible defense when facing threats [38, 39]. These defense responses have been classified as either pattern-triggered immunity (PTI) or effector-triggered immunity (ETI) [39]. PTI consists of the sensing of pathogen-associated molecular patterns (PAMPS) by the plant, triggering a myriad of defense responses, such as callose deposition and induction of the salicylic acid (SA), jasmonic acid (JA) or ethylene (ET) pathways [40], whereas ETI depends on the recognition of pathogen-effectors, deploying highly race or strain-specific responses, culminating in the hypersensitive response [41]. The deployment and magnitude of these defense responses are highly energetically dependent, shifting energy (ATP and NADH) from general metabolism towards defense responses, which might cause an unbalance in plant growth and development [42]. Plant responses to herbivory, including that of phloem-feeding insects, are complex and rely on heavy cross-talk among defense pathways [43]. In general, wound responses, caused by chewing, induce JA-dependent genes, whereas phloem-feeding induces SA-dependent gene cascades [44]. The induction of the SA-dependent pathway by phloem-feeders overlaps that of pathogenic signatures, mainly due to the bacterial community present in these insects’ stylets, which is secreted into the plant [45]. Conversely, the JA and ET pathways overlap during plant defense responses, where the ET pathway is involved in volatile production and release to attract natural enemies, such as parasitoids [46]. The JA/ET pathways are also negatively correlated with the induction of the SA pathway, meaning that induction of one will repress the other [39]. The influence of both beneficial organisms, as well as of pests and pathogens on plant defense can, therefore, be determined by measuring changes in the expression of specific defense marker genes.

When colonized with endophytes, plants have been reported to acquire an enhanced defensive capacity in several cases [33, 34]. This has been shown for plants colonized by arbuscular mycorrhizal fungi (AMF) [47], as well as plants colonized by *Trichoderma harzianum* Rifai (Ascomycota: Hypocreales) [48]. This induced systemic resistance (ISR) consists of a state of systemic protection, closely associated with induction and priming of defense pathways, allowing endophytic plants to perform better under stress conditions [33, 49]. *Beauveria bassiana* has been shown to induce plant defense responses in date palm (*Phoenix dactylifera* L. (Arecales: Arecaceae)) and grapevine (*Vitis vinifera* L. (Vitales Vitaceae)) [50], but little is known of this enhanced defensive capacity by EF. Some level of plant defense pathway reprogramming in thale cress (*Arabidopsis thaliana* (L.) Heynh. (Capprales: Brassicaceae)) and maize (*Z*. *mays*) by *B*. *bassiana* has been reported [51, 52], but the potential influence of these plants' defense responses on natural enemies have not yet been elucidated. Furthermore, the possible mechanisms behind the changes in plant defense responses when treated with *B*. *bassiana* and further challenged by pests have yet to be determined.

This study aimed to investigate how the inoculation of *Vicia faba* seeds with the EF *B*. *bassiana* influences the host-choice of the aphid parasitoid *A*. *colemani* in a two-choice arena set-up. Furthermore, we observed the development time and emergence of adult parasitoids as well as differences in the plant defense responses to aphid infestation. Given our previous positive results on aphid fecundity in a similar model system [15], we hypothesized that aphid nymphs exposed to EF-treated plants would influence parasitoid development and ovipositioning preference. As mentioned, few have reported on plant defense reprogramming by *B*. *bassiana*. We hypothesized that there might be changes in the plants’ initial defense response to the aphids in the EF-treated plants compared to non-fungus treated control plants similar to other symbiotic microorganisms. We have evaluated this by measuring changes in the expression of the specific marker genes PR1 and PR2, involved in the SA pathway, as well as ERF-1, involved in the ET pathway.

Confirmation of endophytic colonization is time-consuming and not always successful [53, 54, 69]. As has been the case in other studies [55, 56], endophytic colonization was therefore not confirmed in this study but assumed based on the observed significant interactions between fungus, plant and insects.

## Materials and methods

### Preparation of fungal spore suspension

To initiate the fungus culture, *B*. *bassiana* strain GHA (BotaniGard) was spread on potato dextrose agar (PDA). After 2–3 weeks (20°C, darkness) the fungus was harvested in a 0.2% Tween20 solution using an inoculation spreader (Sarstedt A/S) to scrape the surface of the fungus culture as described in [15]. The spore suspension was prepared by centrifuging the collected fungal material two times at 4,000 rpm (3076 x g) for 4 minutes, thereafter adjusting the concentration to 10^7^ spores/ml by spore counting in a Fuchs-Rosenthal counting chamber. The percentage of germinating spores was checked the following day by observing more than 200 spores to ensure it was satisfactory (above 90%).

### Preparation of *V*. *faba* plants

*Vicia faba* seeds (cv. ‘Vertigo’) were surface disinfected before sowing by being soaked in 70% ethanol for two minutes, rinsed in sterile water, submerged for two minutes in 1% sodium hypochlorite and finally rinsed three times in sterile water as described in [15]. To assess the surface sterilization efficacy, 1 mL of the final rinse water was plated on PDA medium. Excess amounts of seeds (ca. 400 total) were prepared. Following surface disinfection, half of the seeds (ca. 200 seeds) were soaked in 250 ml spore suspension (10^7^ spores/ml) of *B*. *bassiana* for two hours, while the other half (control seeds) were soaked in 0.2% tween20. Then, three to four seeds were sown in 0.5l pots in autoclaved (25 minutes at 121°C) soil (1:1:1 peat:soil:sand). Following the emergence of seedlings, plants were thinned to one plant per pot. The plants were kept in a greenhouse at 20±3°C in net covered cages (h85cm x w65cm x d75cm). An automatic irrigation system provided water and nutrient for the plants twice daily. After three weeks of growth (five sets of leaflets–growth stage 38 [57]), plants were set to be used in the choice-parasitism experiment and for gene-expression sampling.

### The aphids

A rearing colony of *Aphis fabae* Scopoli (Hemiptera: Aphididae) was kept in a separate net covered cage (h85cm x w65cm x d75cm) in a greenhouse at 20±3°C. To obtain a uniform aphid cohort, similar-sized (second and third stage) aphids were taken from the rearing colony and used in the parasitism experiment following commonly used procedures [58–61].

### The parasitoids

Upon receiving mummies of *Aphidius colemani* from Borregaard BioPlant ApS, they were placed in a plastic container with access to sugar water (70% sucrose). From the onset of adult emergence, mummies were allowed to hatch and the adults to mate for 48 hours with access to sugar water. The newly emerged mated females (48 hours old) were subsequently used in the choice-parasitism experiment.

### Choice-parasitism experiment

**Fig 1:**
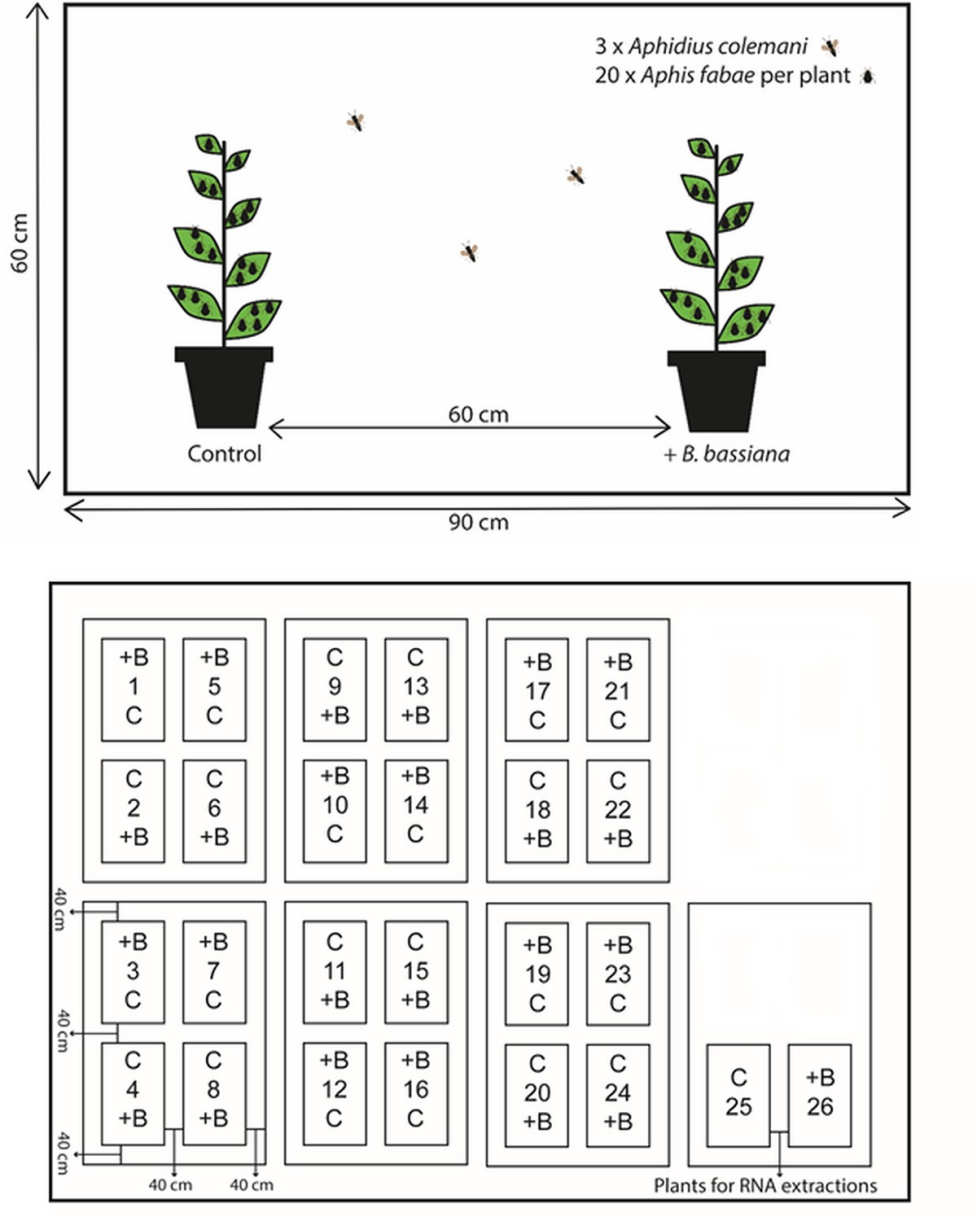
Illustrations of the set-up used for the choice-parasitism experiment. Top: Illustration of a choice-arena showing the spacing of plants and dimensions of the cages. Bottom: Schematic illustration of the greenhouse where “+B” denotes *B*. *bassiana* inoculated plants and “C” the control plants. Numbers refer to individual cage numbers. Cage 25 and 26 contained all plants for RNA extractions (twelve per treatment).

The experiment was set up as a choice experiment in ‘choice arenas’ in a greenhouse at 23±3°C and a 16:8 LD period, supplemented with artificial light in dark periods. Each arena consisted of an insect mesh cage (Golden.Y at Amazon.co.uk, Inc.) with dimensions 60x60x90 cm. Twenty-four cages (replicates) were set up simultaneously. Each arena had two *V*. *faba* plants spaced circa 60 cm apart, a seed-inoculated plant in one end and a control plant in the other. The positioning of the plants and the arenas in the greenhouse is shown in Fig 1. Cages were arranged in a block structure designed to avoid light and temperature gradients, and to limit interference between plants of different treatments. Twenty similar-sized mixed second and third stage aphid nymphs were placed on each plant with a fine painters brush and allowed to settle for 48 h before introducing the parasitoids. The aphids were distributed onto the first and second parts of leaflets of each plant. Three newly emerged and mated female *A*. *colemani* were then introduced in the center of each cage and allowed to parasitize for 24 h, after which the aphid carrying leaves were cut off and placed in designated Petri dishes (100 mm diameter) (one for each plant). To maintain leaf vigor, leaf bases were placed in fitted wet OASIS foam, which was rewetted every 1–2 days. Petri dishes with aphids were kept in a climate chamber at 23±1°C, 70% RH and 16:8 LD and inspected every 1–3 days for the formation of mummies and the subsequent emergence of adult parasitoids. On each inspection day, adults were counted and removed. Dry or otherwise damaged leaves were replaced with fresh leaves of the same treatment, and aphids transferred with a painter’s brush following common procedures [21, 62, 63]. Nymphs (unquantified) produced by observed aphids were removed during inspections. Inspections were continued until it was certain that the emergence of parasitoid adults had ceased (>25 days).

### Gene-expression evaluation in EF-treated and control plants

Leaf samples were taken 9 am at 0, 24, 48 and 72 hours post aphid infestation (single plants only sampled once), shock-frozen in liquid N, lyophilized, ground and stored at minus 80°C in plastic vials, following the protocol described in [64]. Plants used in the sampling were grown simultaneously and under similar conditions as those in the choice experiment, each plant being exposed to twenty similar sized second and third stage nymphs and presented to parasitoids at the same time points. Three first true leaves (six leaflets) were harvested from plants (biological replicate). The material was harvested in 120 mL screw cap containers. Three biological replicates (from different plants) were independently carried out for each treatment and time-point, and two technical replicates (two reactions with a template from the same plant) of each were considered for measurements of relative gene expression.

Changes in the relative expression of the genes: PATHOGENESIS-RELATED PROTEIN 1 (PR1), PATHOGENESIS-RELATED PROTEIN 2 (PR2), and ETHYLENE-RESPONSIVE FACTOR 1 (ERF-1) were evaluated by two-step RT-qPCR. The changes in expression were normalized against changes in expression of the housekeeping gene ELONGATION FACTOR-α (ELF- α), using the 2^ΔCT^ method [65]. Total RNA was extracted from faba bean leaf samples, using an RNA NucleoMag Kit (744350.1 Macherey-Nagel GmbH & Co. KG) according to the manufacturer’s instructions. First-strand cDNA was synthesized from 1 mg of total RNA using a High activity cDNA Reverse Transcription Kit (4368814 Applied Biosystems) according to manufacturer’s instructions. RT-controls were added to account for gDNA contamination. Primers for the abovementioned target genes were selected from the literature [66–68] (additional information on primer sequences and PCR efficiencies in S1 Table). The qPCR reactions were performed on an Applied BiosystemsR ViiA 7 Real-Time PCR System. These were carried out using 1 μL (10 mM) primer pair, 2 μl cDNA template, 6 μl SYBR green master mix (Quanti Tech SYBR Green kit, Qiagen, GmbH Hilden, Germany) and diluted to a final volume of 12 μl with RNase-free water, following the protocol described in detail in [64]. In the negative controls, RNase free water was used to replace the cDNA template as no-amplification controls (NAC). The qPCR program was as described in detail in [64], following the steps: heating step of 2 min at 50°C, 10 min initial denaturation at 95°C, 40 cycles of denaturation for 15 s at 95°C, annealing for 1 min at 60°C. The fluorescence signal was measured immediately after incubation for 1 min at 60°C following the annealing step, and at the end of the cycles, melting temperatures of the PCR products were determined between 60°C and 95°C.

### Statistical analysis

#### Choice parasitism experiment

Data from the choice-parasitism experiment was analyzed using R [69] with generalized linear mixed models (GLMM) using the package lme4 [70]. The proportion of recovered aphids, mummies and emerged adults were analyzed in three independent GLMMs. The three GLMMs all used a binomial distribution and shared treatment as a fixed effect and cage number (1 to 24) (replicates) as a random factor (*logit* link function). To analyse the average juvenile development time of the parasitoids, we fitted a linear mixed effect model (normal distribution–*identity* link function). We used treatment as a fixed effect and cages as a random factor. Models were validated using R package DHARMa [71] via QQ-plots of residuals and using KS-, dispersion- and outlier tests. To test if the positioning of cages had an effect, we tested the models using ‘block’ (each table of four cages) as the explanatory variable. Block was not significantly associated with our responses and was removed from final analyses. Comparisons of means were done using simultaneous tests for general linear hypotheses using the package multcomp [72]. Calculations of means and standard errors for plotting purposes were done using the delta method in the package car [73]. Unadjusted *p* values for reporting significant differences (*p* value < 0.05) are used throughout the data presentation. SigmaPlot Version 11.0 (Systat Software, San Jose, CA, United States) was used for making plots.

#### Gene expression evaluation

Using R [69] gene-expression data were analyzed using linear models (Expression ~ *B*.*bassiana*) to assess differences in gene expression between *B*. *bassiana* treated and untreated plants within each sampling time-point followed by a type I analysis of variance (ANOVA). SigmaPlot Version 11.0 (Systat Software, San Jose, CA, United States) was used for making plots.

## Results

### Choice parasitism observations

**Fig 2:**
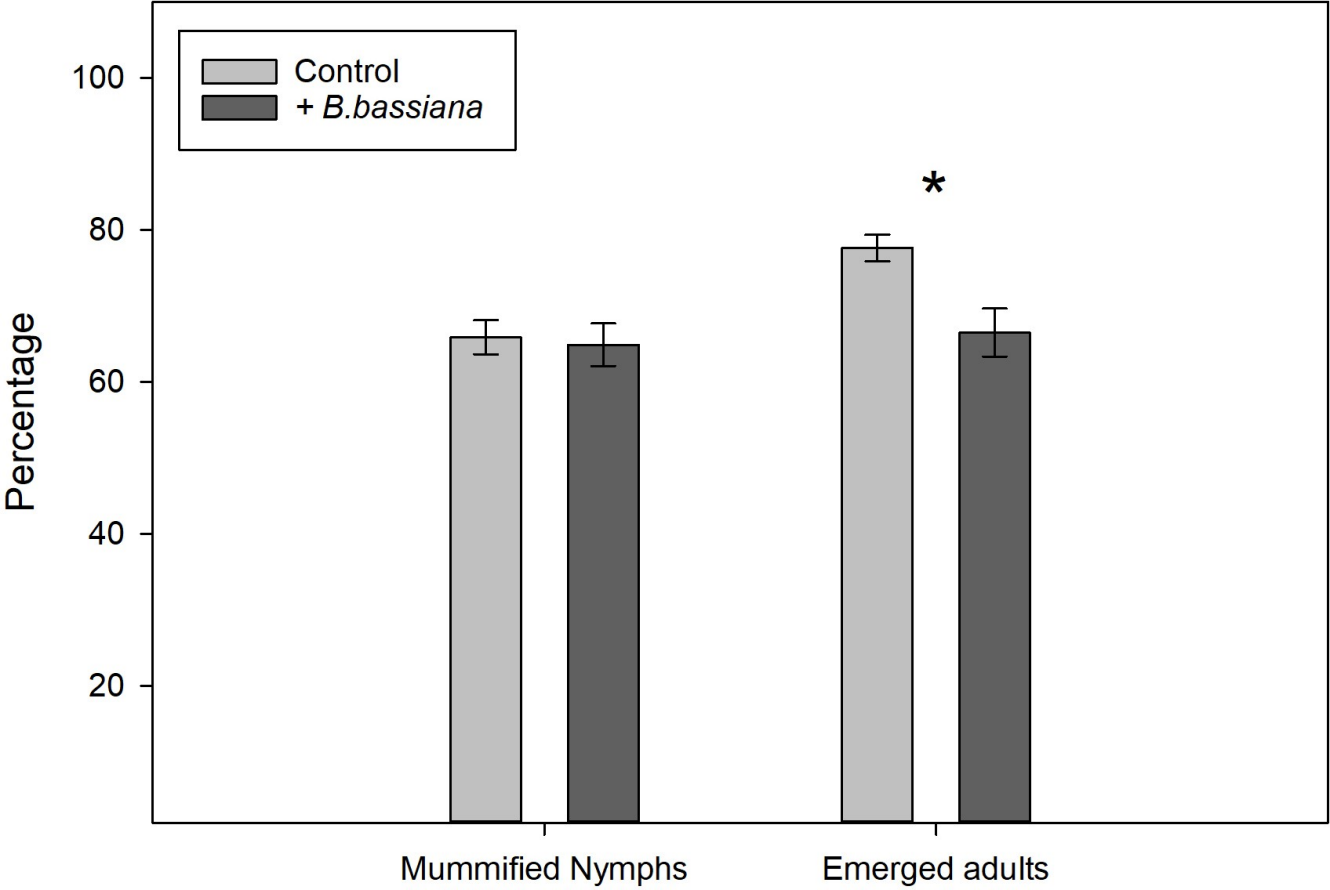
Mean percentage of mummified aphids and emerged adult parasitoids from aphids collected from choice-experiment arenas. The data presented are mean percentages from twenty-four replicates (arenas). Error bars represent standard errors. Statistically significant differences indicated by * (*p* value < 0.05).

The proportion of aphids recovered from the arenas for subsequent observation did not differ between treatments (0.81 for the control and 0.80 for *B*. *bassiana* seed treatment, z-value = 0.33, residual *df* = 45, *p* value = 0.74). The percentage of mummified aphids on inoculated plants and control plants did not differ significantly (66% and 65% respectively, z-value = 0.29, residual *df* = 45, *p* value = 0.77) (Fig 2). However, the percentage of emerging adult parasitoids on *B*. *bassiana* inoculated plants was significantly lower than the emergence in the corresponding control treatment (67% vs. 76%, z-value = 2.6, residual *df* = 45, *p* value = 0.0096) (Fig 2). We found no significant difference in the mean juvenile development time of the parasitoids between the treatments (z-value = 0.16, residual *df* = 45, *p* value = 0.88), the mean development time being 9.1 days for both treatments.

### Gene-expression evaluations in faba bean leaves

**Fig 3:**
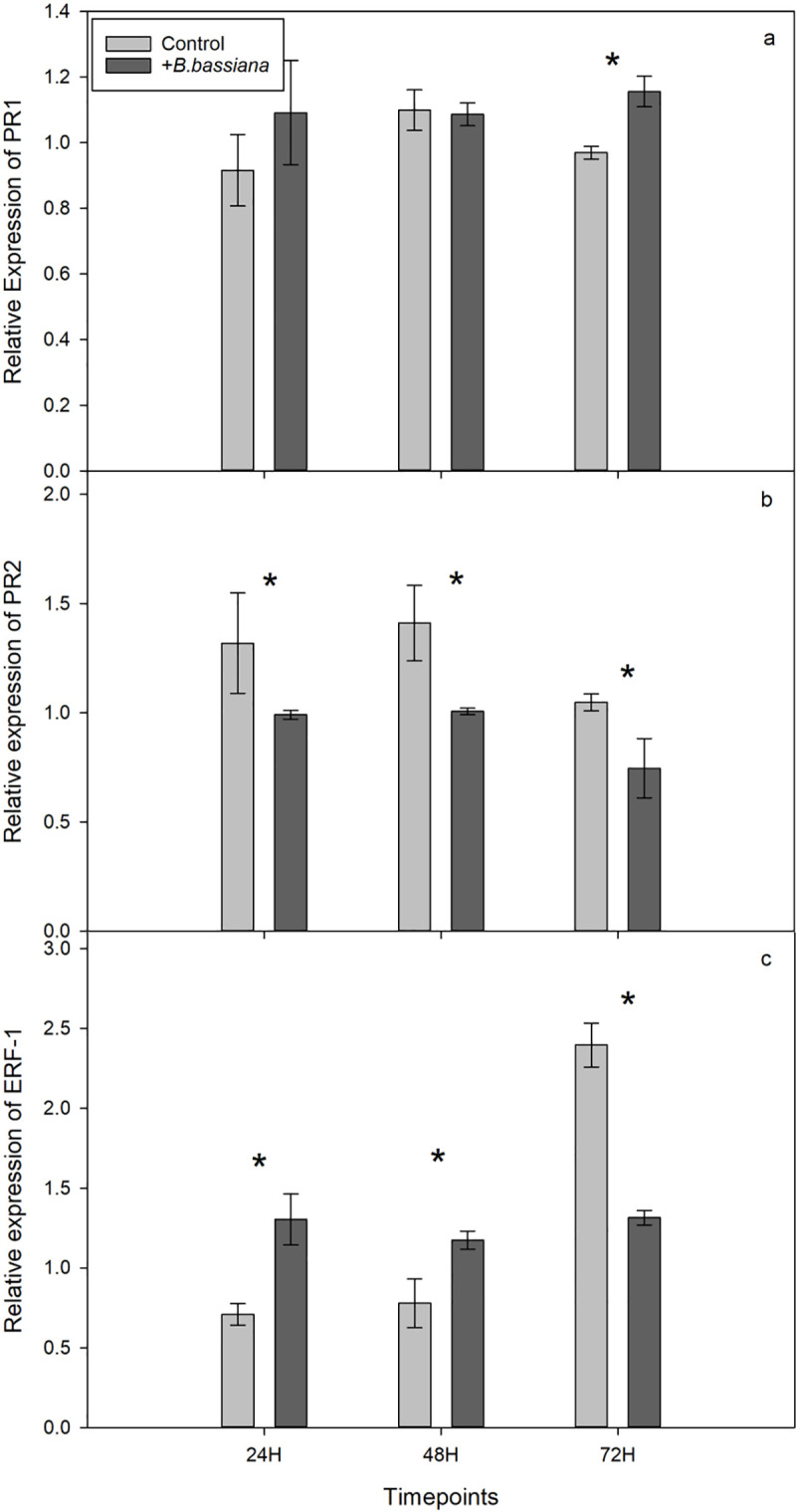
(a) Relative expression of PR1 in *Vicia faba* plants at 24, 48 and 72 hours after *Aphis fabae* infestation. (b) Relative expression of PR2 in *Vicia faba* plants at 24, 48 and 72 hours after *Aphis fabae* infestation. (c) Relative expression of ERF-1 in *Vicia faba* plants at 24, 48 and 72 hours after *Aphis fabae* infestation. Plants were either seed-inoculated with *B*. *bassiana* or not. Statistical significance between treated and untreated plants at each time point indicated by * (ANOVA, *p* value < 0.05, n = 3). Error bars represent ± SE of the mean.

Relative expression of PR1 was significantly higher in *B*. *bassiana* treated plants at 72 h post aphid infestation when compared with control plants (Fig 3A, ANOVA, *p* value = 0.002, F = 43.13, residual *df* = 4). Relative expression of PR2 was consistently lower in *B*. *bassiana* treated plants when compared to control in all time points (Fig 3B, ANOVA, *p* values: 24h = 0.007, 48h = 0.012, 72h = 0.03, F: 24h = 25.8, 48h = 18.66, 72h = 10.5, residual *df* = 4). Relative expression of ERF-1 was significantly higher in *B*. *bassiana* treated plants at 24 and 48 hours post infestation, whereas at 72 h post infestation, expression in control plants was significantly higher than in *B*. *bassiana* treated plants (Fig 3C, ANOVA: *p* values: 24h = 0.03, 48h = 0.03, 72h = 0.01, F: 24h = 9.54, 48h = 10.4,72h = 17.47, residual *df* = 4).

## Discussion

We have shown the effects of a plant-fungal interaction obtained via seed treatment without focusing on the location of the fungus inside the plant. Very little is known of how EF colonize plants [13], and our results emphasize that interactions manifest themselves after simply treating the seed with a spore solution. There are multiple reports of effects on insects where retrieving of the fungus from the host plant failed, maybe due to the limited growth of the fungus within the host plant [13]. Also, methods for screening and quantifying endophytic colonization vary greatly and come with several caveats such as contamination problems [12] and underestimation of colonization [74]. Endophytic colonization has in previous studies been assumed without testing for colonization [55, 56].

In this study, we found a negative relationship between seed-inoculated *B*. *bassiana* and the aphid parasitoid *A*. *colemani*, as the emergence of parasitoids was significantly lower for progenies matured on fungus-inoculated plants. This confirms our initial hypothesis that the development of parasitoids would be influenced by EF-treatment. There are many reports of fungal endophytes generally influencing the development of parasitoids and other natural enemies adversely [4, 5, 59, 75–81]. In most cases, the production of fungal secondary metabolites (alkaloids in general) and subsequent changes to plant chemistry is presumed to be the cause of the observed negative effects. However, symbiotic fungi in plants influence plant metabolism via multiple routes. For instance, mycorrhizal fungi can stimulate plant defense by inducing a cascade of changes in plant metabolism [82, 83].

This is to our knowledge the first report of an antagonistic interaction between a parasitoid and a plant inoculated with EF, as the few studies conducted on interactions between EF-treated plants and parasitoids so far have shown a mostly neutral relationship [35–37, 84]. The one study we found directly comparable to ours (a choice-set-up with *A*. *colemani*), found no significant differences in the preference (expressed as percentage mummification and adult emergence) of *A*. *colemani* for cotton aphids, *Aphis gossypii* Glover, feeding on *B*. *bassiana*-colonized plants compared to non-colonized control plants [36]. However, the large ratio of parasitoids to aphids (100: 20) used in the 24 h parasitization experiment, combined with the unusually low parasitization percentage (approx. 50%) [36], suggests significant interference between the parasitoids, thereby perhaps masking a possible preference and thus making a comparison to our results difficult [36]. We have previously observed a positive effect of endophytic EF on aphids [15], but the majority of research done on endophytic EF and herbivores, including sap-sucking insects, report a negative effect on the insects (i.e. an enhanced plant defensive capability) [10, 12, 13]. Since the success of the intricately associated parasitoids relies on the quality of their host [85], it makes sense to assume some kind of negative consequence on the higher trophic level. A stronger negative effect on parasitoids than their herbivore hosts have previously been demonstrated in studying *Epichloë* spp. endophytes [80]. Furthermore, *B*. *bassiana* applied directly for pest control has previously been reported to have a negative relationship with parasitoids acting indirectly through lowering the quality of the aphids/diminishing the propensity to oviposit in the hosts and lowering parasitoid emergence [26]. We did not observe a difference in the development time of the parasitoids between treatments and our reported development times fit well with previous results at similar temperatures [86]. Our hypothesis that parasitoid development would be influenced by EF treatment was not confirmed in this case. The parasitoid exposed aphids in our study were kept on detached leaves rather than on whole plants, and we expect that using whole plants could have increased the effect on parasitoid larval development had it been possible to do so [13]. We did not test for other parasitoid parameters such as sex ratio and weight of newly hatched adults. Further experimentation in this regard will allow us a better understanding of the influence of EF-treated plants on parasitoids.

We found that newly hatched female *A*. *colemani* did not discriminate between aphids on plants inoculated with *B*. *bassiana* and on control plants, as the percentage of mummified nymphs did not differ between treatments. This does not confirm our hypothesis that EF-treatment would influence parasitoid ovipositioning preference. The small instar nymphs used were allowed to settle on experimental plants for 48 hours before the introduction of parasitoids. By increasing the initial exposure time, the influence on nymphs could become greater, but it would also allow nymphs to reach a suboptimal stage for parasitoid performance. The percentage of parasitized aphid nymphs fits well with a previous report for the parasitization of *A*. *fabae* by *A*. *colemani* [87], confirming that our set-up functioned well, allowing parasitoids to parasitize, but not increasing the incidences of interference, including superparasitism.

As mentioned above, many endophytes, in general, are known to affect parasitoids negatively. It has been found that older female parasitoids can distinguish between endophytic and non-endophytic plants thus enhancing the success of their progenies, whereas younger females cannot do this [79]. We used young females with no prior experience with EF-treated plants, which perhaps explains why they did not make a choice regarding a host plant. However, it should be considered that the example given involved *Epichloë* endophytes, which have a different mode of action than our system (fungal alkaloids). Even so, these results confirm the notion, especially on higher trophic levels, that each scenario must be evaluated on a case-by-case basis, and effects on higher trophic levels are very hard, if not impossible, to predict.

We found differences in defense gene marker expression, and thus, in the plant response to aphid infestation when comparing *B*. *bassiana* treated and control plants, and this is consistent with a previous study using endophytic *B*. *bassiana* [50]. Gene-expression results revealed a delayed induction of PR1 at 72 h post infestation in *B*. *bassiana* treated plants, contrasting with results of previous studies using other endophytic fungi, where induction of PR1 occurred faster than in control plants [43, 64]. Furthermore, PR2 expression was consistently lower in *B*. *bassiana* treated plants in all time-points. Interestingly, as PR1 is a known SA-dependent marker, and PR2 a partly-dependent SA gene [38], these results suggest that there was a repression of the SA-response to aphid infestation in *B*. *bassiana* treated plants. This repression might be related to the changes in the expression of the ET-pathway gene evaluated in the current study. ERF-1 expression was higher in *B*. *bassiana* treated plants at 24 and 48 h post infestation, becoming higher in control plants at 72 h post infestation. As this gene is an ET receptor, these results might indicate that *B*. *bassiana* treated plants had a stronger induction of the ET pathway at an earlier time-point than control plants, which would, in turn, lead to the SA-pathway repression as discussed above [46]. Previous studies have shown that ISR by endophytic fungi, namely AMF, is related to the JA/ET pathways [49]. Thus, the combined changes in the SA- and ET-pathway markers shown here suggest that there might be a response similar to ISR conferred by the EF, which has not previously been shown to occur in response to EF treatment. Even though only a limited set of genetic markers were analyzed in the current study, these results confirm our hypothesis that the changes in the plants’ initial defense response are similar to that seen for other symbiotic microorganisms. Moreover, it indicates that further detailed evaluations of the defense pathways in *B*. *bassiana* inoculated plants after pest challenges are of interest for future studies. This is especially relevant, as a previous report of the defense reprogramming in thale cress (*A*. *thaliana*) showed that, resistance establishment was correlated with fungal pathogen infections but not with pest challenges [51]. Additionally, whether the higher ET induction in *B*. *bassiana* inoculated plants evidenced in this study would lead to differential ET or volatile production remains to be addressed in future studies. This is especially relevant, as changes in production would change how natural enemies perceive signaling by the plant. As we did not observe a difference in the choice-behavior of the parasitoids, this aspect needs further investigation on a chemical level, by evaluating volatile production after aphid infestation. Furthermore, instances of ISR by AMF have been shown to be related to changes in metabolism, as well as differential production of antioxidants by the plant [88, 89]. Due to the similarities in the changes to the plants’ defense responses evidenced in the current study, research on changes of antioxidant production induced by EF treatment might also be of interest in future studies.

In conclusion, these results showed initial compatibility between the two higher trophic organisms, *B*. *bassiana* and *A*. *colemani* (no avoidance), although the lower emergence of parasitoid progenies lead to a diminished second generation. From an ecological viewpoint, this could cause a negative outcome for the parasitoid and would be a disadvantage in inoculative biocontrol strategies where the control relies on the progeny of released individuals. However, in an inundative biocontrol strategy, the rate of parasitism for the first generation might lead to sufficient control of an aphid infestation. This is one of the very few reports on changes in plant defense responses following inoculation of plants with an entomopathogenic fungus. The plant defense responses documented here showed similar patterns to those shown after colonization by other symbiotic microorganisms. This indicates a response similar to induced systemic resistance, which has not previously been shown for an interaction between an entomopathogenic fungus and a plant.

## Supporting information

S1 Table Additional information on the primer sequences used in this study. Additional primer information.
(DOCX)

S1 Dataset Data on choice parasitism, juvenile development and gen expression used for analysis. (XLSX)
