# Supplementary material for: Influence of the plant interacting entomopathogenic fungus Beauveria bassiana on parasitoid host choice-behavior, development, and plant defense pathways
Source: PLoS One. 2020 Sep 14;15(9):e0238943. doi: 10.1371/journal.pone.0238943 (PMC7489556; doi:10.1371/journal.pone.0238943)
Supplement: S1 Table — Additional primer information. (DOCX) [file pone.0238943.s001.docx]

**Supporting information S2 table.** Additional information on the primer sequences used in this study

| Gene | Primer sequence (5′–3′) | Slope | PCR efficiency (%) | Target gene |
| --- | --- | --- | --- | --- |
| *VfELF1A-f*  *VfELF1A-r* | GTGAAGCCCGGTATGCTTGT  CTTGAGATCCTTGACTGCAACATT | -3.36 | 98.4 | *Elongation factor 1-α ^1^* |
| *VfPR1-f*  *VfPR1-r* | CAGTGGTGACATAACAGGAGCAG  CATCCAACCCGAACCGAAT | -3.20 | 105 | *Pathogenesis-related protein* 1 ^1^ |
| *VfPR2-f*  *VfPR2-r* | CCAATGGGTACAAAGAAACG  AAACCAAGTAACCAATGAAAGG | -3.40 | 96.8 | *Pathogenesis-related protein 2 ^2^* |
| *VfERF1-f*  *VfERF1-r* | CTAATGAATATCAAGGTCCTAATTCATC  CATTGCATGCCATTTGATATTCTTCAAC | -3.14 | 108 | *Ethylene response factor 1 ^3^* |

1. Gutierrez N, Giménez MJ, Palomino C, Avila CM. Assessment of candidate reference genes for expression studies in *Vicia faba* L. by real-time quantitative PCR. Molecular Breeding. 2011;28(1):13-24. doi: 10.1007/s11032-010-9456-7.

2. El-Komy MH. Comparative Analysis of Defense Responses in Chocolate Spot-Resistant and -Susceptible Faba Bean (*Vicia faba*) Cultivars Following Infection by the Necrotrophic Fungus *Botrytis fabae*. The Plant Pathology Journal. 2014;30(4):355-66. doi: 10.5423/PPJ.OA.06.2014.0050. PubMed PMID: PMC4262288.

3. Zhou Y, Andriunas F, Offler CE, McCurdy DW, Patrick JW. An epidermal-specific ethylene signal cascade regulates trans-differentiation of transfer cells in *Vicia faba* cotyledons. New Phytologist. 2010;185(4):931-43. doi: 10.1111/j.1469-8137.2009.03136.x.
